# Host defense against fungal pathogens: Adaptable neutrophil responses and the promise of therapeutic opportunities?

**DOI:** 10.1371/journal.ppat.1009691

**Published:** 2021-07-29

**Authors:** Allison K. Scherer, Alex Hopke, David B. Sykes, Daniel Irimia, Michael K. Mansour

**Affiliations:** 1 Division of Infectious Disease, Massachusetts General Hospital, Boston, Massachusetts, United States of America; 2 Department of Medicine, Massachusetts General Hospital, Boston, Massachusetts, United States of America; 3 Harvard Medical School, Boston, Massachusetts, United States of America; 4 Center for Engineering in Medicine and Surgery, Department of Surgery, Harvard Medical School, Boston, Massachusetts, United States of America; 5 Shriners Burns Hospital, Boston, Massachusetts, United States of America; 6 Center for Regenerative Medicine, Massachusetts General Hospital, Boston, Massachusetts, United States of America; 7 Harvard Stem Cell Institute, Cambridge, Massachusetts, United States of America; Duke University School of Medicine, UNITED STATES

Neutrophils are the most abundant innate immune cells in the bloodstream and play critical roles in defending the body against invasive bacterial and fungal infections. While short lived, neutrophils rapidly deploy a variety of mechanisms to eradicate microbial pathogens including phagocytosis, the release of antimicrobial peptides, the production of reactive oxygen species (ROS), the formation of neutrophil extracellular traps (NETs), and the secretion of cytokines and chemokines. Additionally, neutrophils maintain a wide breadth of roles in homeostasis, including the clearance of necrotic tissue, and in disease where they become a source of damaging inflammation [1,2]. Because of this functional plasticity in both homeostasis and infection, neutrophils are hypothesized to exist as unique, diverse subsets of heterogeneous populations of cells.

Heterogeneity can be defined in a variety of ways, including differences in the host cellular compartments, maturation stage, and through functional distinctions, which allow cells from the same population to perform different tasks. Various groups have utilized these definitions to describe distinct cell populations in the adaptive and innate immune cell branches. In the adaptive immune compartment, T- and B cell populations are well characterized to have unique subtypes with distinct functionality. Furthermore, clonal heterogeneity in these cells can be identified through the expression of highly unique T- and B cell receptors. More recently, functional and phenotypic heterogeneity has emerged for innate immune cells, including macrophages, monocytes, and dendritic cells, which was assessed through sophisticated functional assays and comprehensive analysis of surface markers (e.g., cytometry by time of flight (CyTOF)) at a single-cell level. Of particular interest and relevance is the heterogeneity found in monocytes, where subpopulations are shaped under the influence of developmental, health and disease states, cell origin, transcriptional regulators, and extrinsic inflammatory signals [3]. Another potential avenue for describing heterogeneity includes epigenetic reprogramming or “trained immunity.” Here, innate immune cells, such as monocytes, undergo epigenetic and metabolic reprogramming in response to a stimulus [4], and, upon restimulation, there is a decreased inflammatory response. This trained immunity is a long-term adaptation [5] and represents a possible mechanism by which neutrophil subtypes could also be reprogrammed [6,7]. Future work will be needed to determine if these mechanisms are effective in neutrophil populations. Here, we review current evidence that neutrophils demonstrate similar forms of heterogeneity as defined by these criteria.

Characterizing these potential subpopulations would provide several key benefits to both laboratory and clinical applications. The identification and characterization of heterogeneity in neutrophils would be a critical advance in our understanding of neutrophil biology. Theoretically, patients with innate immune deficiencies could be managed through enrichment of granulocyte populations identified to be particularly effective against specific bacterial or fungal infections. However, neutrophils can also cause significant off-target damage to the host, and the potential for different subpopulations to cause immunopathology will need to carefully be examined. To that end, the identification and characterization of these potential subpopulations are important areas of research, and the development of tools to define these subpopulations may have direct consequences on our understanding of both neutrophil biology and patient therapy.

It has been suggested that neutrophils differentiate into subsets defined by discrete functional and phenotypic characteristics such as variance in cell surface markers, transient localization, and differences in effector function [8–12]. While single-cell functional assays to define committed activity are currently impractical, single-cell transcriptional differences have shed light on potential neutrophil subpopulations. Here, we explore mounting evidence for neutrophil heterogeneity in (1) developmental homeostasis; and (2) subsets that arise during inflammation or invasive infections. We highlight how characterizing these subpopulations may lead to advancements in host-directed therapies to combat bacterial and fungal pathogens.

## Neutrophil heterogeneity during development

**Fig 1:**
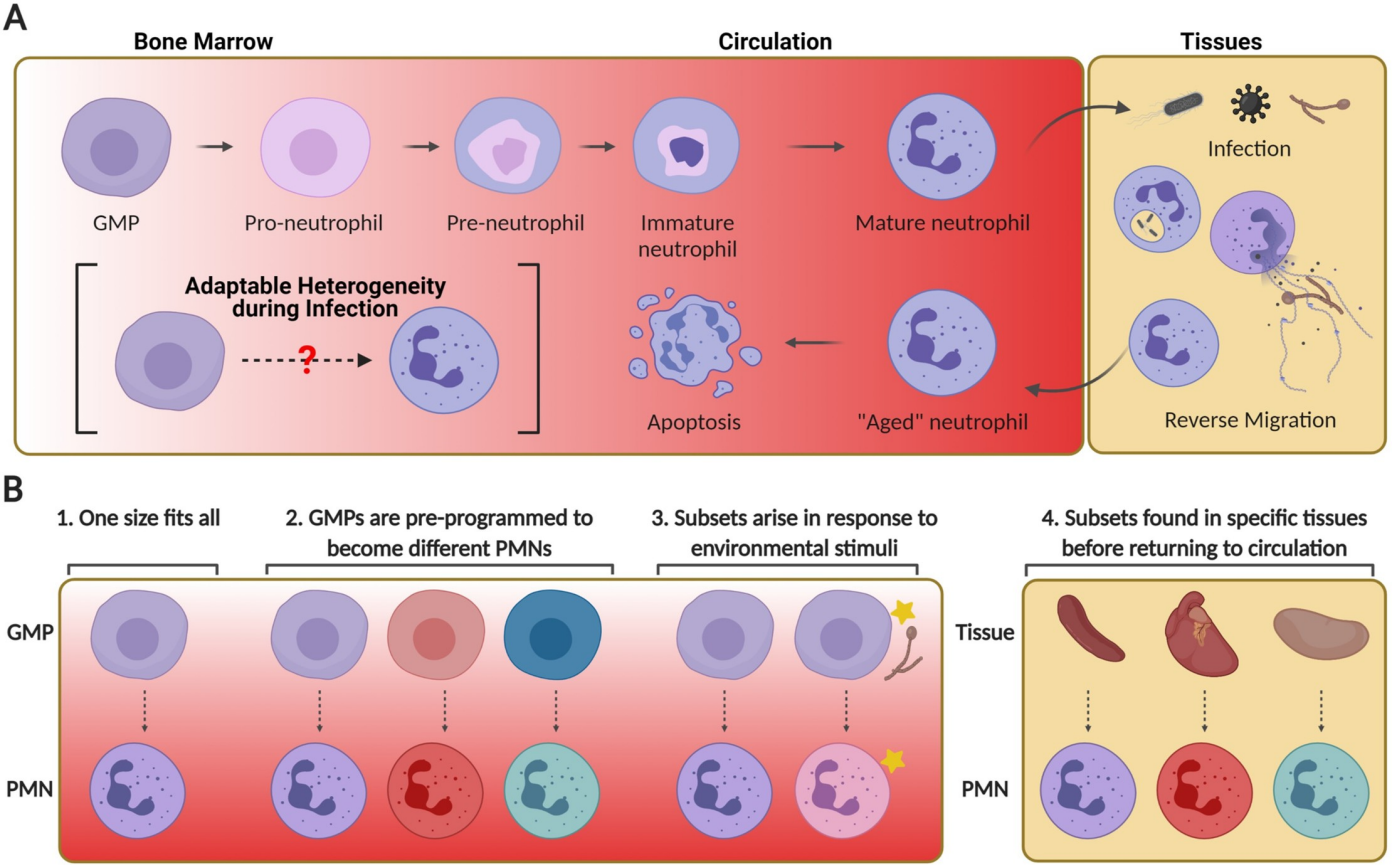
Schematic of proposed areas of neutrophil heterogeneity. **(A)** GMPs proliferate in the bone marrow after which they mature in circulation (bloodstream), and mature neutrophils hone to specific tissues or remain in circulation. **(B)** Potential areas for neutrophils to sort into separate subpopulations of cells. Created with BioRender.com. GMP, granulocyte monocyte progenitor; PMN, polymorphonuclear leukocyte.

During homeostasis and disease, neutrophil heterogeneity arises from the different developmental stages within the bone marrow. There, granulocyte monocyte progenitors (GMPs) give rise to proliferative myeloblasts, premyelocytes, and myelocytes as well as banded or segmented neutrophils and mature and circulating neutrophils as illustrated in Fig 1A (adapted from [13]). Of note, hematopoietic stem cell (HSC) heterogeneity has been demonstrated in response to conditions of physiologic stress, including infection [14]. Here, myeloid-based HSCs expand to meet the demand for specific cell lineages to combat pathogens [14,15]. These maturing myeloid populations are controversial, recognized as being subjective as based on morphological and histochemical observations and not necessarily indicative of specific functional or transcriptional differences [13]. Recent single-cell transcriptomic studies combined with corresponding multiparametric flow analyses in humans [16,17] and mice [16] illustrate the existence of early, intermediate, and late neutrophil precursor cell stages, each with unique gene and transcription factor signatures [13] supporting neutrophil heterogeneity.

Mouse neutrophil populations have now been characterized by single-cell transcriptomics during homeostasis and bacterial infection; authors were able to link specific cells from mouse peripheral blood, spleen, and bone marrow with previously described stages of neutrophil development based on classical cell morphology [16]. Neutrophils demonstrate transcriptionally distinct subpopulations with differences in interferon-stimulated gene expression, lipopolysaccharide-mediated signaling pathways, as well as others [16]. Here, unsupervised clustering demonstrated 8 distinct clusters, which were classified as G0 through G4 and G5a through G5c cells [16]. G0 through G4 subsets included cells originating and differentiating from the bone marrow including GMPs. G5a through G5c cells mainly came from the peripheral blood and were classified as mature neutrophils [16]. Authors also demonstrated that this differentiation and maturation pattern seen in the G0 through G5 cell subsets occurred in an organized and regulated fashion [16]. Additionally, recent work demonstrates unique neutrophil subsets in healthy tissue, suggesting tailored function to specific tissue niches [18]. Together, these indicate that previously identified populations through morphology and other physical characteristics do appear to be consistent with transcriptional differences.

## Neutrophil heterogeneity in malignancy

In the setting of inflammatory conditions, such as cancer, the number of neutrophils in circulation increases, and the phenotype of the cells is altered [19]. Neutrophils may increase metastatic seeding by inhibiting natural killer cells or expedite extravasation, and neutrophilia is associated with poorer outcomes in cancer patients [20]. Pathogenesis is thought to be related to increased expression of matrix metalloproteinases allowing spread of tumor cells [20]. Tumor-associated neutrophils have been characterized as either N1 or N2 cells in mice [1,20] and in humans [21]. N1 and N2 cells are likely more associated with the G5b subset of neutrophils; however, the tumor microenvironment alters the transcriptome of the neutrophils, significantly suggesting that another area of neutrophil heterogeneity may exist in response to cancer-specific stressors [16]. N1 neutrophils are characterized by the production of pro-inflammatory cytokines, potent antitumor capacity, and hypersegmented nuclei [1,22]. N2 neutrophils accumulate at the tumor site, are cancer promoting, and have an immature phenotype [1,22]. It is not clear whether N1 and N2 cells share a common origin site [1]. Recently, N1 and N2 neutrophils were generated *in vitro* from primary human neutrophils, demonstrating that this phenomenon is not solely restricted to mice [21]. Interestingly, in the setting of infection, the anti-inflammatory N2 neutrophils were unable to kill the parasite *Leishmania donovani* as effectively as N1-polarized neutrophils [21].

## Neutrophil heterogeneity in combating bacterial and viral pathogens

Some circulating neutrophils respond better to specific bacterial pathogens than other neutrophils, termed “competitive phagocytosis,” although this phenomenon has not been correlated with any specific Polymorphonuclear leukocyte (PMN) markers to identify phenotypes associated with differences in phagocytosis [1]. However, human mature neutrophils infected with *Helicobacter pylori* will differentiate into N1-like cells marked by nuclear hypersegmentation [22,23]. Neutrophils taken for single-cell RNA sequencing after host challenge with *Escherichia coli* demonstrate reprogramming of neutrophil populations so that cells are primed for antimicrobial functionality without losing overall heterogeneity [16]. Differences in expression of the NADPH oxidase complex genes and granule-related genes were also seen within *E*. *coli*–primed neutrophil subpopulations [16]. Taken together, there are distinct functional and phenotypic differences between subsets, including antimicrobial properties. However, it remains unclear if these subpopulations are deliberately selected during bacterial infection.

Similarly, neutrophils in the context of viral infection, including bunyavirus, hepatitis C and B viruses, and influenza, promote antiviral immune responses [24]. In influenza, apoptotic neutrophils release epidermal growth factor promoting antigen presentation, which are critical for CD8+ T cell–mediated protection [25]. Most recently, Coronavirus Disease 2019 (COVID-19) has demonstrated increased neutrophil numbers and dysregulated immune response in severe cases of disease [26]. Single-cell RNA sequencing and single-cell proteomics of patient samples reveal that inflammatory monocytes with interferon-stimulated gene signatures were higher in mild COVID-19 disease, whereas severe disease was marked by increased neutrophil precursors as seen during emergency myelopoiesis [26]. Furthermore, subsets of neutrophils taken from patients with severe COVID-19 demonstrated impaired oxidative burst [26]. Additional research is needed to elucidate the mechanisms controlling innate immune cell dysregulation during COVID-19 infection and the roles different neutrophil populations play during aspects of this viral infection.

## Discrete functional and phenotypic neutrophil characteristics in response to fungal infection

Neutrophil heterogeneity has also been noted in response to fungal pathogens. Specific neutrophils subsets characterized, for example by Dectin-2 receptor expression, were proposed to acquire augmented identification and elimination of specific fungal species through *trans*-differentiation. In the presence of invasive fungal pathogens, neutrophils *trans*-differentiated expressing antigen-presenting machinery. These hybrid polymorphonuclear leukocyte–dendritic cells (PMN–DCs) were better able to eliminate and activate T cells in response to fungi [27]. During blastomycosis and aspergillosis, PMN–DCs associated with fungal cells killed them more efficiently than their canonical neutrophil counterparts and had increased expression of pattern recognition receptors [27]. Identifying these types of neutrophil subpopulations in response to fungi through effector function characterization will allow better granularity of neutrophil heterogeneity.

Recent work examining single-cell transcriptomics of murine neutrophils challenged with *Cryptococcus neoformans* has also revealed a method of describing neutrophil heterogeneity in the context of a pulmonary infection. Here, authors analyzed a previously published dataset of *C. neoformans*-infected mice and identified 2 neutrophil subsets specific to *C. neoformans* infection [28]. In the first, an oxidate stress signature identifies neutrophils (Ox–PMN) that likely interact with *C. neoformans* directly to generate ROS [28]. The second subset demonstrates a prominent cytokine gene expression profile from neutrophils (Cyt–PMN) that indirectly respond to *C. neoformans*-derived ligands and interact more closely with other host immune cells for a coordinated response [28]. Additionally, gene expression patterns illustrate unique spatial localization of these neutrophil subsets to the lung, in the parenchyma, or vasculature depending on the subset [28]. These data suggest that different neutrophil subsets work in tandem to coordinate the host response and clear *C. neoformans* lung infection [28].

The process of characterizing neutrophil subpopulations with enhanced effector function will require effective tools, ideally those which can go beyond traditional individual function assays and simultaneously examine multiple functions. An example of this multifunctional analysis is the use of a novel assay characterizing neutrophil swarming behavior. The assay, which was used to characterize neutrophil swarming responses to *Candida albicans*, provides a platform that can simultaneously examine multiple critical host–pathogen events including neutrophil motility, signaling, and antimicrobial function, including NET release, all of which help determine the effectiveness of the swarming response. The assay provides granularity into pathogen responses, allowing analysis of overall fungal growth/restriction as well as discrete events such as time to fungal germination and hyphal escape from neutrophil control [29]. The increased complexity of the swarming assay offers many benefits for studying neutrophil function, although it still offers a simplified view of neutrophil responses and fails to recapitulate critical events, such as extravasation and migration through tissues, which can alter and activate neutrophils. The design of future microfluidic devices will build upon these benefits while incorporating even more complexity to fully reflect *in vivo* conditions and allow the detailed dissection of differences in neutrophil subpopulation behavior.

If the neutrophils were equally equipped to respond to fungal challenge, a “one size fits all” category (Fig 1B), then we would anticipate an equal neutrophil response to fungal pathogens. However, in human neutrophils isolated from healthy, cirrhotic, and transplant patients, whole population heterogeneity can be seen in response to *C*. *albicans*. Here, the swarming assay was leveraged to dissect the abilities of circulating neutrophils to mobilize and contain *C*. *albicans* growth where healthy neutrophils swarmed and effectively restricted fungal growth while cirrhotic patient neutrophils failed to contain *C*. *albicans*. In contrast, neutrophils from transplant patients displayed more heterogeneity between patients in the ability of their swarms to restrict fungi [30,31]. We found that human disease alters the whole population of circulating neutrophils independent of disease severity; however, one limitation of the assay is the difficulty in parsing out individual neutrophils to follow their response to the target over time. More robust assays will be required to parse out if specific subsets of neutrophils are better equipped to undertake the complex and diverse tasks required to contain or kill fungal pathogens and if changes to subsets are involved in differences we see in patient neutrophil function. These sophisticated functional assays will be critical for further identifying and characterizing neutrophil subpopulations and would augment molecular profiling through single-cell RNA sequencing.

## Therapeutic potential for patients with fungal disease and perspectives

Infections continue to cause significant mortality in a wide variety of patient populations, highlighting the need for novel therapeutic strategies to combat these pathogens. With the emergence of antibacterial and antifungal resistance and the threat posed by simultaneous coinfection with multiple pathogens, alternative therapies will need to include strategies that can be tailored to highly specific contexts to maximize beneficial outcomes. The identification of immune-based therapies has, therefore, become more appealing in the last several decades. Unique challenges are present in preserving the defensive characteristics of neutrophils while simultaneously disabling their ability to bring about damaging inflammation [32]. By selecting certain phenotypic or functional attributes of specific neutrophils such as nuclear morphology, NET formation, buoyancy, surface markers, and immunomodulatory function, we may be able to generate neutrophils that are optimally prepared to respond to specific stimuli [32]. A clinical trial in which neutropenic patients with probable infection were supplemented with daily transfusions of granulocytes from donors was conducted [33]. Although there was no overall benefit to the patients in the granulocyte transfusion arm, post hoc analysis suggests that patients that received a higher dose of granulocytes had better outcomes than those that received a lower dose [33]. These patient-based data are promising as these suggest that specific numbers of granulocyte subpopulations lead to improved immune-based therapies. Given evidence of specific human neutrophil subpopulations [30,31], these data suggest that the higher dose is effective because either patients require a large number of granulocytes overall or the higher dose may have contained a degree of specific neutrophil subpopulations.

To further investigate the idea of granulocyte transfusion as therapy, mice were irradiated to render them neutropenic and then transfused with GMPs transduced with a conditionally immortalized murine system [34,35]. The GMPs transfused into the neutropenic mouse model were then challenged with heat-killed *C*. *albicans* where differentiated neutrophils were able to identify and phagocytose the yeast [35]. These data support the use of progenitor-derived neutrophils for *in vivo* challenges to identify functionally distinct neutrophil populations. Our future directions are to characterize the molecular mechanisms responsible to differences and identify markers to select for these subpopulations. We will examine how specific populations of GMPs, and, ultimately, their corresponding neutrophil counterparts, can be used to provide protection to patients at risk of invasive fungal infections.

## Conclusions

Defining heterogeneity for neutrophils has been previously challenging for technical reasons related to these short-lived cells. With advancements in single-cell transcriptomics and conditional immortalization of GMP cells, exploring the different aspects of neutrophil heterogeneity has become feasible. Future clinical trials examining GMP transfusions and resulting neutrophil subsets will be promising. Through specific targeting of neutrophil subsets, the potential of a new class of cellular therapeutics for patients with a wide range of inflammatory disorders from malignancy to infection is possible.
